# Categorization of Fetal Heart Rate Decelerations in American and European Practice: Importance and Imperative of Avoiding Framing and Confirmation Biases

**DOI:** 10.14740/jocmr2166w

**Published:** 2015-07-24

**Authors:** Shashikant L. Sholapurkar

**Affiliations:** Department of Obstetrics and Gynaecology, Royal United Hospital Bath NHS Trust, Bath, UK. Email: s.sholapurkar@nhs.net

**Keywords:** Cardiotocography, Electronic fetal monitoring, Fetal heart rate decelerations, Intrapartum fetal monitoring, Intrapartum fetal surveillance, Confirmation bias, Framing bias, Anchoring bias

## Abstract

Interpretation of electronic fetal monitoring (EFM) remains controversial and unsatisfactory. Fetal heart rate (FHR) decelerations are the commonest aberrant feature on cardiotocographs and considered “center-stage” in the interpretation of EFM. A recent American study suggested that the lack of correlation of American three-tier system to neonatal acidemia may be due to the current peculiar nomenclature of FHR decelerations leading to loss of meaning. The pioneers like Hon and Caldeyro-Barcia classified decelerations based primarily on time relationship to contractions and not on etiology *per se*. This critical analysis debates pros and cons of significant anchoring/framing and confirmation biases in defining different types of decelerations based primarily on the shape (slope) or time of descent. It would be important to identify benign early decelerations correctly to avoid unnecessary intervention as well as to improve the positive predictive value of the other types of decelerations. Currently the vast majority of decelerations are classed as “variable”. This review shows that the most common rapid decelerations during contractions with trough corresponding to peak of contraction cannot be explained by “cord-compression” hypothesis but by direct/pure (defined here as not mediated through baro-/chemoreceptors) or non-hypoxic vagal reflex. These decelerations are benign, most likely and mainly a result of head-compression and hence should be called “early” rather than “variable”. Standardization is important but should be appropriate and withstand scientific scrutiny. Significant framing and confirmation biases are necessarily unscientific and the succeeding three-tier interpretation systems and structures embodying these biases would be dysfunctional and clinically unhelpful. Clinical/pathophysiological analysis and avoidance of flaws/biases suggest that a more physiological and scientific categorization of decelerations should be based on time relationship to contractions alone irrespective of shape or descent time as indeed proposed by pioneers like Hon and Caldeyro-Barcia. Such meaningful categorization, apart from being a scientific necessity, could improve the practical performance of three-tier FHR interpretation systems and possibly application of dependent complementary techniques like fetal ECG/pulse oximetry/computer-aided analysis, thus facilitating future progress in the field of intrapartum fetal monitoring.

## Introduction

Cardiotocography (CTG) during labor is the commonest medical procedure in the western world and also very extensively studied. Notwithstanding, the interpretation of fetal heart rate (FHR) patterns during labor remains the most controversial and problematic issue in obstetrics. Out of many FHR parameters, the FHR decelerations seem most common and important but also complex to interpret. Leading experts from New Zealand and USA [1] concluded that the FHR decelerations are “center-stage” in interpretation of CTG. They emphasized that the clinicians should be trained to understand the (correct) physiologic mechanisms of decelerations and the patterns of FHR change that indicate progressive loss of fetal compensation [1]. Clearly the physiological mechanisms proposed should be correct and scientific for proper interpretation. To facilitate separation of benign from potentially pathological decelerations, their categorization into early, late and variable has been practiced for half a century. This categorization was the so-called “low lying fruit” quickly picked up by pioneers like Hon and Caldeyro-Barcia based on simple observational studies [2-4]. They classified FHR decelerations primarily based on their time relationship to contractions. In general the “low lying fruit” tends to have major correlation to the outcomes being studied. On the other hand, the subsequent clinical trials and some of the evidence-based medicine (EBM) far down the line may sometimes be left looking for marginal differences or benefits [5]. There is fair evidence that British practice had “early decelerations” (possible main cause - head compression) as the majority until 2007 [6, 7]. However, the North American and the current British National Institute for Health and Clinical Excellence (NICE) guidelines state that “variable” decelerations are most common and “true uniform early” decelerations are very rare [8-10]. This view is generally accepted in the rest of Europe. The current categorization is based on expert opinion only and not on any evidence or correlation to fetal condition [11]. A high quality American study (Editor’s choice) found no correlation of American three-tier system to neonatal acidemia [12]. It concluded that the current peculiar nomenclature of FHR decelerations seems responsible for poor correlation with fetal status and thus loss of meaning. A recent prominent review even forwarded a somewhat confused argument that the current obsession with categorization of FHR decelerations is unhelpful and should be abandoned [13], a very minority view at present. It would indeed be a major loss to abandon the “low lying fruit” picked up by the pioneers. Sartwelle and Johnston (2015) argue that during medico-legal proceedings, the evidence from electronic fetal monitoring (EFM) should be considered invalid and inadmissible, based on the “Daubert doctrine” which excludes “junk science” from the courtrooms [14]. They make a strong reasoned argument for a “change in course or abandonment of the ship (i.e. EFM)” [14]. This commentary debates whether the aforementioned disillusionment with EFM may be foremost due to a flawed/dysfunctional categorization of decelerations (center-stage) due to the phenomena of “anchoring/framing/confirmation biases”. The presence and the type of FHR decelerations are very often deterministic in the three-tier classification of CTGs. The American three-tier system of CTG interpretation has been found unhelpful in actual practice [12] because more than 80% of all CTGs fall in category II, mainly as a result of almost all FHR decelerations being (wrongly?) classed as “variable” (cord compression) but almost none as “early” (benign). This analytical review makes a case for a fundamental reform to adopt a more physiologic and scientific categorization of FHR decelerations which would serve as a robust foundation for the three-tier interpretation systems. The three-tier system itself or proposing an alternative “proven” system is not the subject of this review. However, with a change of course in the right direction, more reliable three-tier systems of interpretation should evolve.

## Confirmation and Anchoring/Framing Bias

Acknowledged experts Parer and Hamilton proposed that framing and confirmation bias may occur during CTG interpretation in some clinical situations [15]. “Confirmation bias” occurs when we selectively focus upon evidence that supports our beliefs, while ignoring more comprehensive evidence that disproves these ideas [16, 17]. It is closely related to “framing (anchoring) bias” which is the tendency to create coherent initial picture without examining all available information [16, 17]. Nickerson pondered whether confirmation bias persists because it has some misconstrued functional value. It could be (wrongly) believed to provide some benefits more important than an attempt to determine the truth in an unbiased way in particular situations [16]. However, framing and confirmation biases are incompatible with scientific pursuit and in the end harmful to clinical practice and patient safety. Confirmation bias is ubiquitous [16, 18]. It has been claimed that experts are similarly prone to these biases as laypeople and the main difference may be in the quantity of arguments people can muster [18]. There can be confirmation biases on both sides of any argument which can be viewed as a “division of cognitive labour” and arguing these in a deliberative and interactive context can lead to valid conclusions or closer to the scientific truth [18].

## History of Categorization of FHR Decelerations

Edward Hon in the USA in his pioneering work described three FHR deceleration patterns based on whether they had their onset at the beginning (early decelerations), 20 - 30 s after beginning (late decelerations) of contractions or if onset time was variable (variable decelerations) [2, 3]. He hypothesized that the early decelerations may be result of head compression and those with variable time relationship to contractions may be due to cord compression although his classification was not “etiological” [2, 3, 19]. The rapid decelerations during contractions (early timing) were later called “type I dips” by Caldeyro-Barcia who suggested alterations in cord blood flow and fetal hemodynamics in addition to head compression as possible etiological factors [4, 19]. Early decelerations were fairly common and found in 12%, 19% and 27% of all labors [19]. Majority of experts in the UK classified all decelerations starting at the beginning of contraction and recovering before the end of contraction as “early” (irrespective of rate of descent) [6, 20]. This made “early decelerations” to be the majority in British obstetric practice over many decades [6, 7, 21, 22]. The etiology of decelerations would always remain putative and possibly multifactorial with one of the causes predominant. Experimental head compression mimicking caused by contractions has been actually demonstrated to cause rapid decelerations during human labor and also supported by observations in twin and breech labors [2, 4]. On the other hand, “cord compression hypothesis” is mostly putative except in cases of overt cord-prolapse where the decelerations are often quite deep, prolonged with late recovery (very different from the common decelerations observed in labor). Sometime in the late 20th century, the American classification of decelerations seems to have become primarily “etiological” despite the many potential pitfalls. Animal experiments showed that artificially induced cord occlusion produced immediate rapid decelerations. Hence, all decelerations with rapid descent were presumed to be due to cord compression even though head compression could also cause rapid decelerations [23]. Thus all rapid decelerations were by definition called “variable” even though majority of them started early during contractions with nadir corresponding to the peak of contraction (early timing). This paradoxically made “early benign decelerations” extremely rare in the recent American practice. Early pioneers had quite quickly observed that decelerations with early timing were benign and those with late timing were likely to be associated with fetal hypoxemia and this represented “low lying fruit” in terms of strength of correlation. FHR decelerations can be said to be of two main types, one due to benign parasympathetic (vagal) reflex and the other due to hypoxic (chemoreceptor) vagal reflex or direct suppression of myocardium in later stages [23]. The clue to differentiating this is in the “timing” rather than “shape” since hypoxia during contraction has a lag time to develop or worsen [23]. FHR decelerations which start recovering immediately after the peak of contraction (early timing) are very unlikely to have hypoxic component and hence it would be important to appropriately recognize them as benign (“early”).

## British and Australian Definitions of Early and Late Decelerations

The current British guidelines defined early and late decelerations to be “uniform” in shape based on Hon’s work (personal correspondence) [8, 24]. But this seems a misinterpretation of Hon’s description of “pattern of uniform time relationship to contractions” [6]. True uniform early and late decelerations (same in depth and duration) as envisaged [25] do not occur, cannot be found and hence fictitious [26]. Secondly, “early” and “late” decelerations are required to be “gradual” (bell shaped) [25]. However, decelerations that look gradual on American CTG (paper speed 3 cm/min) would not look gradual on British CTG (paper speed 1 cm/min). Only relatively shallow decelerations (up to 20 bpm amplitude) will look gradual on British CTG [6]. The Australia New Zealand guidelines also mistakenly define early and late decelerations as “uniform” (same in depth and duration). They also (inexplicably) postulate that “early decelerations” occur between cervical dilatation of 4 - 7 cm and are associated with decreased baseline variability [27], not proposed by any other national guidelines. This may be because Hon [28] used ring pessaries of 3 - 6 cm diameter in his experiments to produce head compression associated decelerations (larger pessaries could be impractical) and that gradual/shallow decelerations (flawed definition?) would be associated with reduced baseline variability. These definitions embodying framing biases/errors need to be corrected for any chance of useful clinical application.

## Pathophysiology of Variable Decelerations

**Figure 1:**
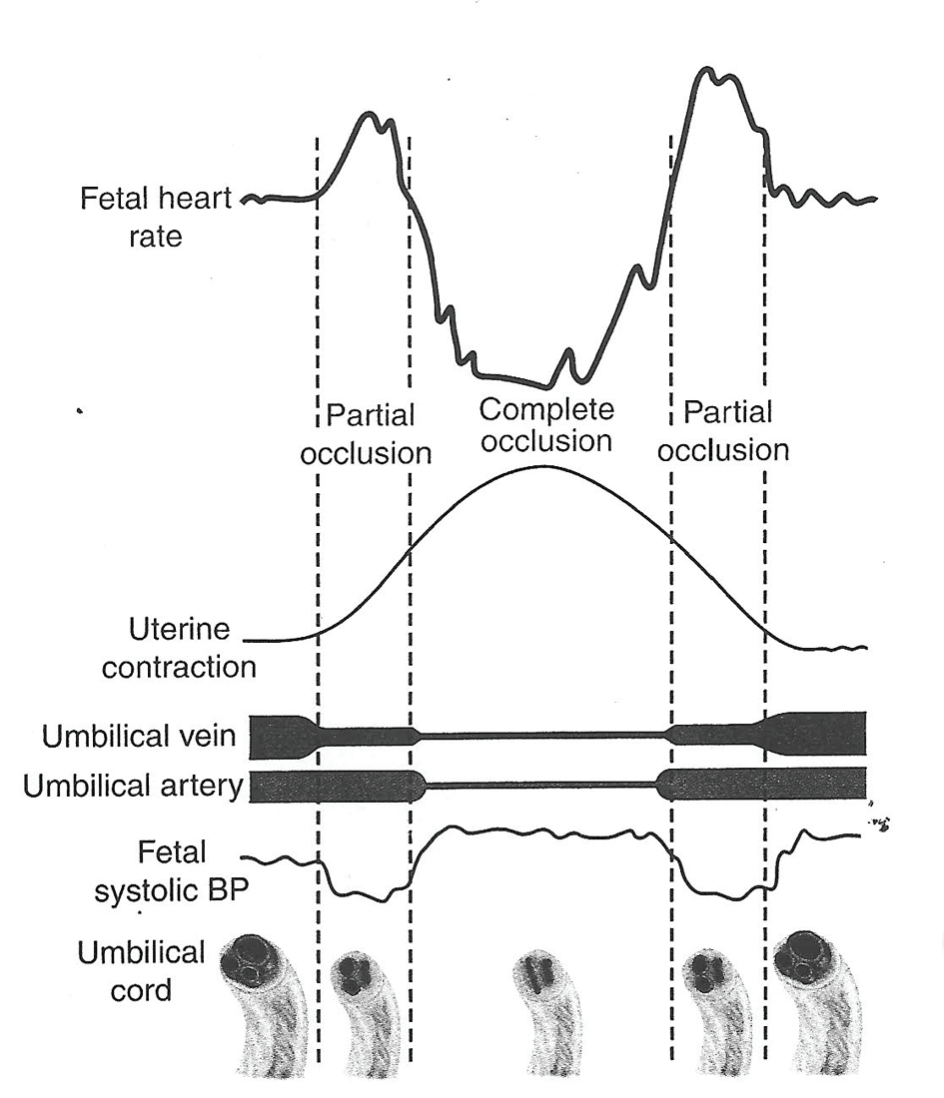
Hypothesis of cord compression and variable deceleration [25, 27, 29, 30] (thankfully reproduced from OJOG 2013;3:362-370, open access) [23]. This hypothesis has major fallacies. Complete cord compression has been postulated for these most common decelerations (CTG paper speed 3 cm/min). The FHR recovery commencing at the height of contraction (where umbilical arterial and venous occlusion is unrelieved) cannot be explained. Instead the deceleration depicted seems consistent with “direct” or “pure” vagal reflex (head compression).

**Figure 2:**
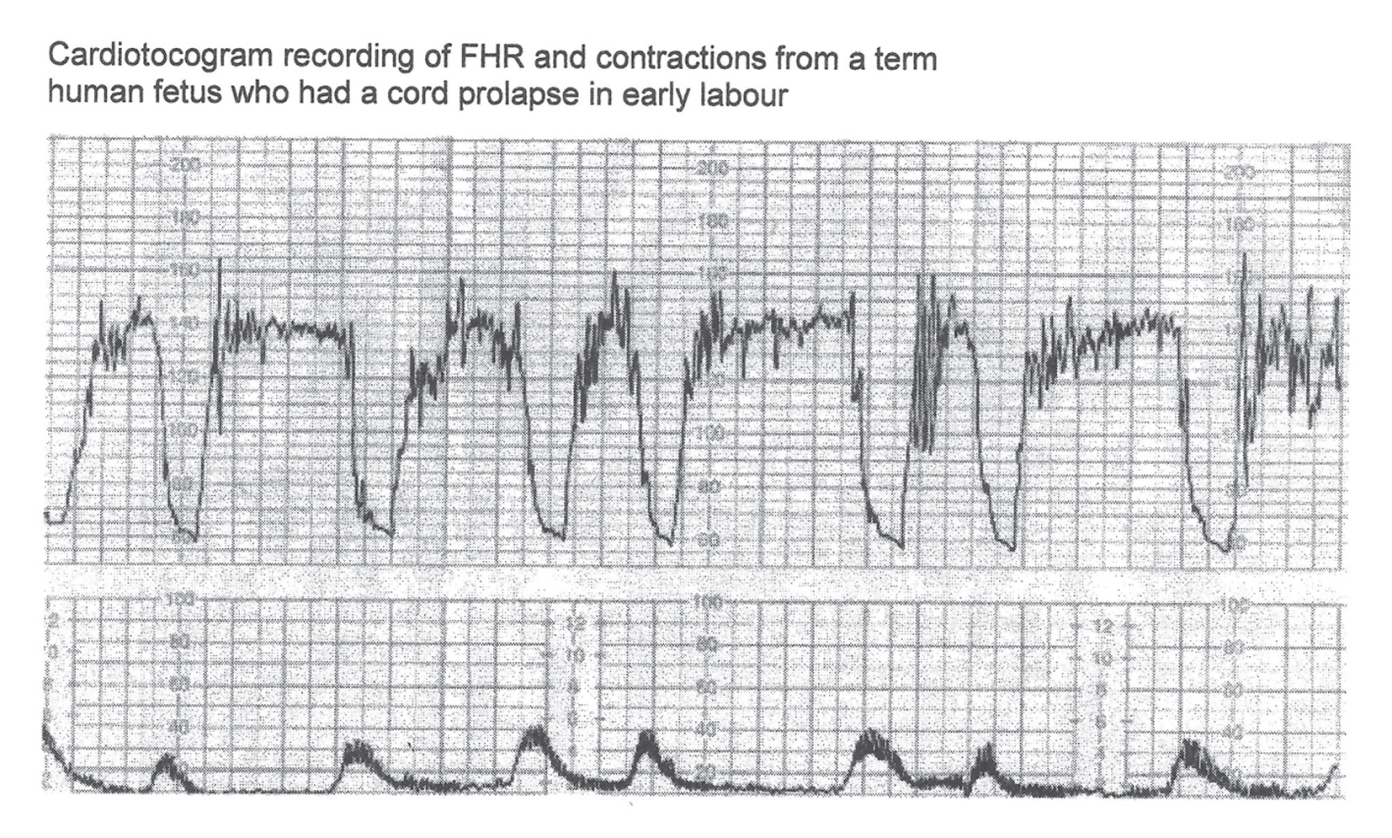
CTG in a case of cord prolapse showing decelerations (mistakenly) classed as “variable” (reproduced with thanks from Westgate et al, AJOG, 2007) [1]. Although these decelerations “look” rapid (paper speed 1 cm/min), the “descent time” is well over 60 s.

**Figure 3:**
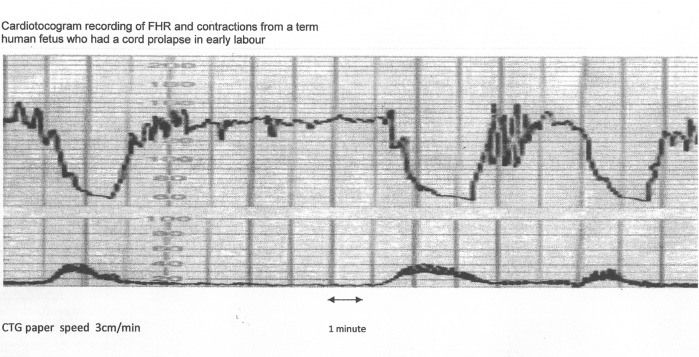
The same CTG from Figure 2 shown with American CTG paper speed of 3 cm/min. Note that the “descent time” is well over 60 s. Hence, based on American definitions [10, 11], these decelerations are “late”, demonstrating that rate/time of descent does not reliably predict etiology.

Variable decelerations (also called cord compression pattern) currently constitute the largest group. Majority of decelerations in fact have “early” timing with trough corresponding to peak of contractions but these are defined as “variable” because of rapid descent time (< 30 s) [10, 11]. The commonest pathophysiological mechanism proposed is the “cord compression - baroreceptor reflex” [25, 27, 29, 30]. However, “chemoreflex” (hypoxemia) seems the main additional mechanism confirmed from animal studies [1, 23]. A critical evaluation suggests that the “cord compression - baroreceptor/chemoreceptor hypothesis” seems incompatible with the commonly observed rapid decelerations (Fig. 1). This is because it cannot explain the recovery of FHR starting at the peak of contraction where umbilical arterial occlusion is un-relieved or indeed maximum. Furthermore, a careful analysis of the chemoreflex mechanism [1] reveals interesting contradictions. If fetus starts developing hypoxemia early during the contraction phase (drop in uteroplacental perfusion or cord compression), then the hypoxemia will not be relieved at the height of contraction, but only the rate of worsening of hypoxemia may slow down (Fig. 2, 3). The hypoxemia (and hence the deceleration) would start recovering much later during relaxation phase when the umbilical “venous” compression is relieved. Only at this later point the recovery of FHR deceleration would be expected to commence. Several animal studies also confirm that the FHR decelerations from cord compression start to recover only after the release of compression [23]. The example given by New Zealand and American experts [1] in a case of known cord prolapse (compression) to illustrate “variable decelerations” demonstrates that these decelerations are very different (deep and prolonged) from the commonly observed decelerations in labor (Fig. 2, 3). It shows that the slope of decelerations slows down after the peak of contraction but the zenith is reached much later during relaxation phase (descent time > 30 s) and the recovery is complete well after the contractions. Thus based on American definitions [10, 11], these decelerations due to cord compression would be considered to have slow descent (gradual) and classed as “late” and not “variable”. This example confirms that “descent time” does not reliably correlate with etiology.

Thus it seems improbable that the decelerations whose trough corresponds to peak of contractions could be because of cord compression based on pathophysiology and animal experiments (Fig. 1). These would instead be consistent with pure/direct vagal reflex (defined here as not mediated through baro-/chemoreceptors) from head compression as predominant cause amongst multifactorial etiology [23]. The precise mechanism of vagal reflex from head compression is unknown but could be stretch on the brain membranes or some degree of rise in intracranial pressure. However, any hypotheses that these decelerations could be because of reduction in intracranial blood flow leading cerebral anoxia and hence dangerous, have been disproven [19]. Another probable mechanism seems “placental compression” transferring blood (through patent cord vessels) to fetus during contraction phase causing fetal bradycardia through baroreceptor stimulation [6]. This flow would reverse during relaxation phase thus the deceleration would start recovering immediately following peak of contraction. But this again is a completely benign mechanism to explain an early deceleration as opposed to contraction-induced “cord compression” or “hypoxia”. The observation that “amnioinfusion” (temporarily) ameliorates FHR decelerations of “early” as well as “variable” timing may be explained by reduction in placental compression and cord compression respectively.

## Balance of Arguments - Framing/Confirmation Biases - Categorizing FHR Decelerations by Rapid/Gradual Shape

**Figure 4:**
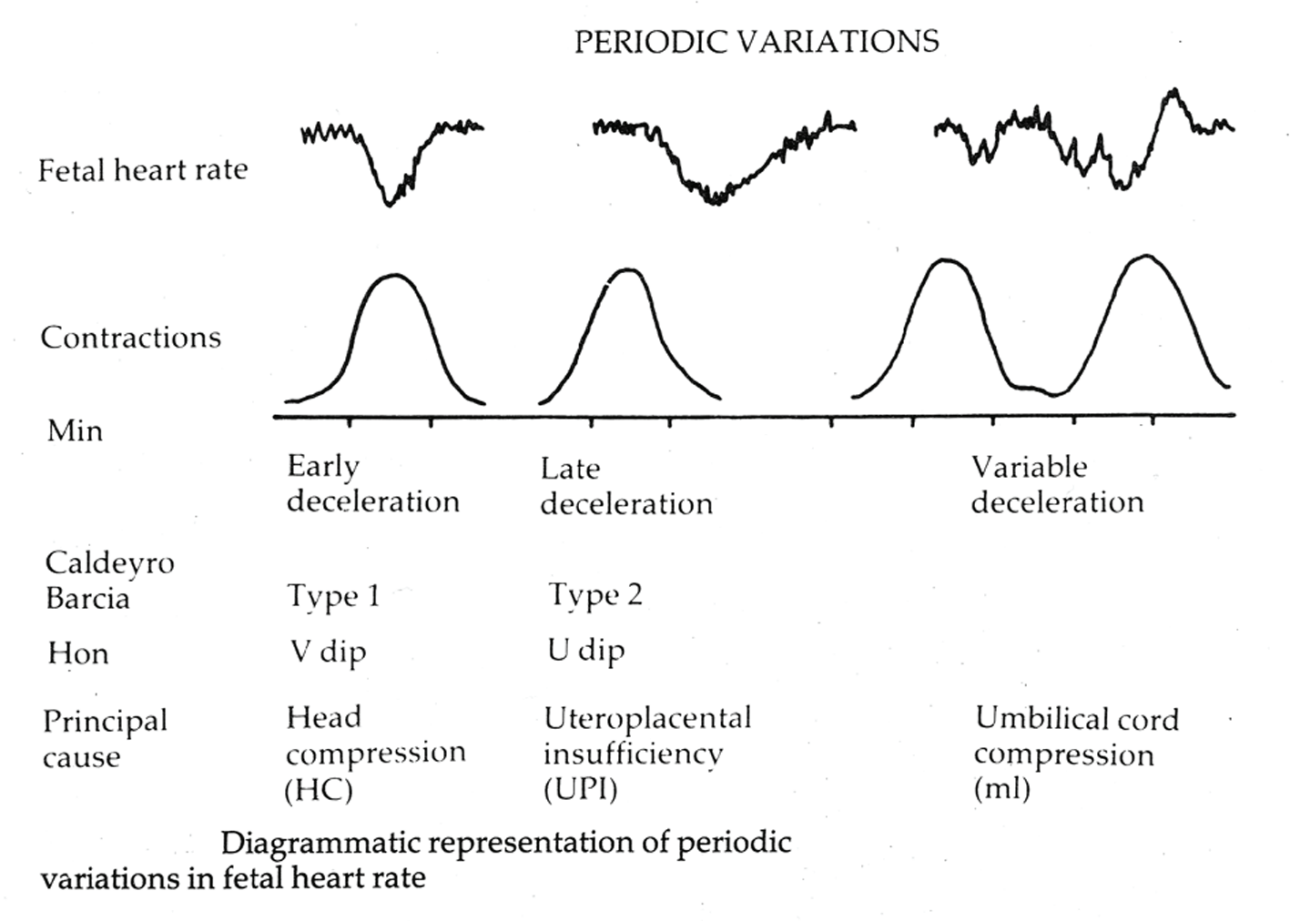
Diagrammatic representation of early, late and variable decelerations as practiced in British Obstetrics before 2007 (reproduced with kind permission from “Principles of Obstetrics” by Bryan Hibbard, 1988) [20]. Note the apparent rapid descent of early decelerations.

Experiments in sheep showed that “clamping” of umbilical cord (partial or complete) was associated with rapid FHR decelerations [1]. Whether that is comparable to what happens during labor contractions or not, the decelerations with rapid descent were assumed to indicate “cord compression” and hence called “variable”. By corollary, decelerations due to head compression (early) were postulated to have slow descent and defined as such. This convenient differentiation soon became ingrained in practice [8-11, 23]. An arbitrary cut-off of 30 s was selected to differentiate rapid from gradual decelerations [10, 11]. Plentiful evidence (see below) and American/British expert observation (Fig. 4) that the head compression also causes “rapid” decelerations tends to be disregarded now (confirmation bias?).

The following points are proposed to support the view that the decelerations should not be classified based on descent time or slope (i.e. rapid - variable; gradual - early or late).

I. The “cord compression - baro-/chemoreceptor” hypothesis proposed [1, 25, 27, 29, 30] for common rapid decelerations during contractions seems to have major contradictions as described above (Fig. 1). II. The American pioneer Edward Hon [2] stated that the “physiologic” drop in FHR at the height of contraction could be attributed to an increase in intracranial pressure (direct or pure vagal reflex). Hon applied pressure to fetal head with a 6-cm pessary and consistently produced a bradycardia similar to that during contractions [28]. III. The group of Caldeyro-Barcia [4] demonstrated that bimanual and vaginal compressions of fetal head resulted in rapid decelerations very similar to type I decelerations. IV. Many American authorities have concluded that decelerations due to head compression have rapid descent [28, 31, 32]. Indeed the leading American experts Ball and Parer [31] remarked that head compression is the likely cause of many so-called “variable” decelerations attributed to cord compression. V. Fairly convincing evidence comes from a commonly observed clinical situation of twin pregnancy presented by Hon [2]. Both fetuses presented by vertex with intact amniotic sacs. The presenting twin whose vertex was dilating cervix consistently had decelerations with contractions while the second twin with head out of pelvis had no decelerations. Hon commented that both twins were subjected to similar compression of placenta (and similar possibility of cord compression) during contractions; and hence this could not have been the cause of decelerations noted. A careful examination of the FHR record provided by Hon [2] reveals that the “descent time” of these decelerations most likely due to head compression is about 10 - 15 s (rapid) and hence would be (wrongly?) classed as “variable” by the current definitions. VI. Hon further stated that the role of head compression in FHR deceleration is supported by the observation that the common contraction-associated rapid decelerations are rare in breech presentation where head is not subjected to pressure in the pelvis [2]. VII. Head compression is common in labor and can be demonstrated to cause decelerations. How can the extreme rarity of “early decelerations” be explained? It can be observed in practice that the recovery time for decelerations is generally equal or longer than the descent time. This would make “early” decelerations (descent time > 30 s) to be always more than 60 - 65 s for no valid reason but just by the very accident of the (flawed) definition contradicting their benign nature [23]. Moreover, contractions lasting for less than 60 s simply cannot have decelerations due to head compression (early) because they cannot accommodate decelerations with descent time > 30 s. Similarly, applying the random attribute of descent time > 30 s to late decelerations could misclassify many which are less than 60 s in duration (descent time < 30 s) [23]. VIII. The fact that all fetuses (although subjected to head compression) do not have early decelerations and that these can be small or big can be explained by the variable degree of head compression and natural variation in biological response. An experiment closer to physiological conditions was carried out by Mocsary and associates who found that early decelerations occur when intracranial pressure reaches 55 mm Hg or more [33].

On the other hand, following seems the evidence possible or arguments generally proposed to support the current categorization of FHR decelerations. I. This is the confirmed current expert view and should be accepted as such. The opposing arguments above are theoretical matters of academic interest only [34]. II. Even if many/most “rapid” decelerations during contractions are mainly because of head compression, now they must be called “variable” by definition. As long as everyone follows the same system, there would be no problem irrespective of any bias/flaws. However, in actual practice this may lead to loss of meaning [12]. III. Nuchal cord and decelerations: The increased incidence of “variable” decelerations in the presence of nuchal cord proves that the “variable” decelerations (as defined) must be because of cord compression. But, when the vast majority of decelerations are defined to be “variable” in the first place, is it surprising that the “variable” decelerations are increased? Does this prove that the prior framing of vast majority of decelerations (whether nuchal cord present or not) as “variable” was correct? [23]. IV. Edward Hon’s pioneering work (1958 - 1968) has proven that head compression causes “gradual” decelerations and all decelerations with rapid descent are due to cord compression. This is actually a common misconception of Hon’s descriptions as already explained in this article [2, 3, 6, 7, 23]. V. There has been a curious and isolated (completely unproven) suggestion that the rapid FHR decelerations with cord compression can be explained by Bazold-Jarisch reflex [35]. This is a controversial reflex which does not play a significant role in short term homeostasis of blood pressure. It characteristically results in quite profound persistent systemic hypotension [36] which simply cannot be the case with vast majority of FHR decelerations in labor. VI. It has been argued that the baseline variability is of paramount importance for fetal hypoxemia/acidemia and categorization of decelerations may not matter (This is incorrect - see next section, Table 1 [12]). VII. Another objection would be the lack of evidence that a categorization focusing on timing of decelerations would improve the detection of fetal acidosis. But there is general lack evidence of effectiveness of most aspects of EFM [9, 10]. This is not an excuse to perpetuate significant framing and confirmation biases which are necessarily unscientific. This would be misapplication of EBM. These biases would undermine all subsequent structures and systems of interpretations embodying them. It has also been argued that the clinical significance of decelerations is not what caused them or the apparent timing of the nadir, but how often they occur, how long they last and how big they get. This is an argument not in favor of current categorization but about disbanding it. Although the size of decelerations would correlate to hypoxemia, it would significantly vary for different types of decelerations.

**Table 1:** A Large Study by Cahill et al (2012) Showing That 91% of Acidemic Babies Displayed Moderate Variability During the 30 Min Before Delivery [12]

FHR pattern	pH ≤ 7.10 (57 newborns)	pH > 7.10 (5,331 newborns)	P value
Moderate baseline variability	91.2%	87.2%	-
Minimal baseline variability	8.8%	12.5%	0.41
Baseline tachycardia	12.3%	4.5%	-
Repetitive late decelerations	15.8%	7.3%	0.05
Repetitive variable decelerations	49.1%	32.5%	0.03
Early decelerations	None	None	-

There were hardly any “early” decelerations in this large study, not because head compression does not cause decelerations, but very likely because of accident of (flawed) definitions. Late decelerations of duration < 60 s (i.e. descent time < 30 s) may have been wrongly classed as “variable”.

It is always possible to find a small number of studies or hypotheses to prove a particular set of conclusions (confirmation bias) but a scientific conclusion should be based on the balance of arguments described above. The categorization of FHR decelerations based on “time or slope of descent” does not correlate to either etiology or fetal condition [23] and is associated with major fundamental contradictions and fallacies. It could be surmised that FHR decelerations are best defined primarily and solely based on time relationship to contractions [2, 3, 6, 23] as proposed in Table 2. Simple (definitions) can be harder (to believe and accept) than the complex (Steve Jobs quoted in Observer).

**Table 2:** Proposed Physiological Categorization of FHR Decelerations [6, 26]

Early decelerations	Recurrent^#^ slowing of FHR with onset early in the contraction and return to baseline at the end of contraction.
Late decelerations	Recurrent slowing of FHR with onset mid to end of contraction and nadir more than 20 s* after peak of contraction and ending after the contraction. If baseline variability is less than 5 beats/min, then the definition would include decelerations less than 15 beats/min.
Variable decelerations	Recurrent slowing of FHR with varying time relationship to the contraction cycle. They tend to markedly vary in shape.

The confusing term “uniform” is avoided. ^#^“Recurrent” - associated with more than 50% contractions [11]. *The 20 s lag-time is based on expert consensus [24] and seems practically useful.

## Simplified Approach to Detect Progressive Loss of Fetal Compensation: Is It Practical?

It has been proposed that the decelerations should be observed until there is evidence of progressive loss of fetal compensation in the form of rising baseline (tachycardia) followed by falling baseline rate and reduced variability [1, 13]. However, most obstetricians have come across babies with significant acidemia/hypoxic ischemic encephalopathy (HIE) following late decelerations alone with normal baseline variability. Moreover, the goal of EFM should be to detect abnormal FHR patterns corresponding to acidemic pH of about 7.10 for expediting delivery and not 7.00 [12]. A recent large high quality study showed that only 8.8% of acidemic babies (pH < 7.10) had reduced baseline variability and only 12.3% had baseline tachycardia (Table 1) [12]. Thus, many fetuses may not necessarily go through proposed sequential “de-compensatory changes” before significant acidemia (pH < 7.10). Moreover, obstetricians would want to deliver the babies in advance of significant risk of neonatal HIE and the policy of waiting for “de-compensatory changes” will need to be rigorously tested before adoption in clinical practice and seems impractical at the current time.

## Conclusions

EFM has been claimed to be a “markedly flawed science” in need of abandonment or radical change of course [14]. FHR decelerations are of critical importance (center-stage) in CTG interpretation [1]. Substantial experimental and clinical evidence presented above suggests that the definitions of decelerations based on rapid/gradual shape or abstract cut-offs of “descent time” seem inconsistent with pathophysiology and putative causation, and leads to loss of meaning. It needs to be debated if this comprises major “anchoring/framing/confirmation biases”. The most common rapid decelerations during contractions with trough corresponding to the peak of contraction cannot be explained by cord compression or hypoxemia, but by direct/pure or non-hypoxic vagal reflex (e.g. head/placental compression). Dysfunctional categorization may lead to disillusionment and even abandonment of longstanding sub-classification into early/late/variable varieties. This may be like throwing the baby out with the bathwater. Obstetricians and birth attendants should debate if there is an imperative to rid of framing and confirmation biases/flaws without necessarily waiting for definitive proof of effectiveness of more scientific categorization of FHR decelerations. Because status quo may amount to knowingly continuing to teach and practice a demonstrably flawed (unscientific) model of pathophysiology and categorization of FHR decelerations. Some evidence may be accrued from re-analysis of data from previous studies. A more physiologic and scientific categorization of decelerations should be based primarily on the “time relationship to contractions” alone as indeed intended by the pioneers Hon and Caldeyro-Barcia [2, 3, 4, 6, 23]. A reformed categorization (Table 1), apart from being a scientific necessity, is likely to have a major favorable impact on CTG interpretation and the utility/evolution of three-tier systems even if not a panacea. Improved visual interpretation of CTG may also facilitate dependent ancillary techniques like fetal ECG, computer-aided interpretation and thus future progress.
